# Spectrally Stable Blue Light-Emitting Diodes Based on All-Inorganic Halide Perovskite Films

**DOI:** 10.3390/nano12172906

**Published:** 2022-08-24

**Authors:** Huidan Zhang, Ying Su, Xulan Xue, Qinghui Zeng, Yifang Sun, Kai Zhu, Weiguang Ye, Wenyu Ji, Xiangyang Leng

**Affiliations:** 1 State Key Laboratory of Luminescence and Applications, Changchun Institute of Optics, Fine Mechanics and Physics, Chinese Academy of Sciences, Dong_Nanhu Road 3888, Changchun 130033, China; 2 University of Chinese Academy of Sciences, Beijing 100049, China; 3 Changchun University of Chinese Medicine, Changchun 130017, China; 4 School of Optoelectronic Engineering and Instrumentation Science, Dalian University of Technology, Dalian 116024, China; 5 Key Lab of Physics and Technology for Advanced Batteries (Ministry of Education), Department of Physics, Jilin University, Changchun 130012, China

**Keywords:** all-inorganic halide perovskite, ligand-assisted reprecipitation (LARP), blue emission, poly(9-vinylcarbazole) (PVK), spectrally stable electroluminescence

## Abstract

Substantial progress has been made in perovskite light-emitting diodes (PeLEDs), but the fabrication of high-performance blue PeLEDs still remains a challenge due to its low efficiency, spectral instability and short operational lifetime. How to produce an efficient and stable blue PeLED is the key to realizing the application of PeLEDs in full-color displays. We herein report a blue PeLED usint the ligand-assisted reprecipitation method, in which phenylethylammonium bromide (PEABr) was used as ligands, and chloroform was used as anti-solvent to prepare blue perovskite nanocrystal films. By increasing the PEABr content from 40% to 100% (The ratio of x% PEABr refers to the molar ratio between PEABr and PbBr_2_), the film quality is highly improved, and the emission exhibits a blue shift. Introducing a poly(9-vinylcarbazole) (PVK) hole transport layer into the device, the PVK layer can not only achieve efficient hole injection, but can also isolate the PEDOT: PSS layer to inhibit the non-radiative recombination of metal halide luminescence layer, reduce surface ion defects and successfully inhibit halide atom migration. Finally, the PeLED presents a stable electroluminescence under different driving voltages without any red shift.

## 1. Introduction

In recent years, all-inorganic metal halide perovskite materials have been widely used in the lighting and display fields due to their high color purity, easy band gap adjustment, narrow-band emission and high fluorescence quantum efficiency, making them ideal candidate materials for the preparation of next-generation light-emitting diodes (LEDs) [1,2,3,4,5,6,7]. As researchers have optimized perovskite composition [8], film quality [9], and device structure [10], great breakthroughs have been made in the external quantum efficiency (EQE) of perovskite light-emitting diodes (PeLEDs) during the past few years. The highest EQE of near-infrared, red and green PeLEDs has exceeded 20% [8,10,11,12,13,14], which is comparable to other commercial quantum-dot and organic LEDs.

As one of the three primary colors of white light, the blue PeLEDs still perform poorly compared with green and red PeLEDs [15,16,17,18], which is due to the fact that the chloride-doped blue PeLEDs are very sensitive to power supply and voltage fluctuations. In other words, the power supply will inevitably suffer voltage fluctuations, which will lead to peak wavelength shift, decreased light efficiency and the inferior reliability of blue PeLEDs [16,19]. This seriously affects its color purity and hinders the commercialization of blue PeLED. Therefore, we still need more effective methods to improve the spectral stability of the devices for chloride-doped perovskite, especially in the applications where the wavelength accuracy and luminous stability of LED are strictly required. Therefore, the blue PeLED need a more stable perovskite emission layer when they are operated in a higher working voltage or a stronger electric field. Therefore, how to improve its EQE and spectralstability is an essential step to accelerate the applications of blue devices.

In general, the realization of blue perovskite luminescence is mainly through halogen-doping component engineering and dimension regulation based on the quantum confining effect. The former tunes the bandgap of the mixed halide perovskite by controlling the bromine and chlorine doping components to realize the emission of blue light from perovskite [20,21,22,23]. The latter is a quasi-two-dimensional perovskite or quantum dot that can form a three-dimensional perovskite cambium by adding an organic ammonium salt or other ligands with long chains [24,25,26]. Yao et al. successfully obtained the blue emission of CsPbBr_x_Cl_3−x_ nanoparticles at 470 nm through halogen-doping component engineering [27]. However, a large amount of Cl^-^ vacancies were easily formed due to the doping of a large amount of chlorine, which introduced deep-level defects, leading to strong non-radiative recombination, hence reducing the luminous efficiency of the blue light PeLEDs [28,29]. Moreover, the migration effect of halogen ions under an electric field will result in the degradation of perovskite films in lighting or stability, then at the electroluminescence (EL) spectrum peak red shift will inevitably occur. Chen et al. adjusted the perovskite structure of CH_3_NH_3_PbBr_3_ by adding different amounts of 2-phenoxyethylamine (POEA) to the CH_3_NH_3_PbBr_3_ precursor solution, resulting in dramatic changes in the photoluminescence (PL) and EL properties of CH_3_NH_3_PbBr_3_ films, changing the emission color from green to blue, and significantly improving the performance of the PeLED [30]. However, the introduction of a large number of long-chain ligands will lead to poor charge transfer ability of perovskite films, affecting the efficiency and brightness of the devices. Moreover, the EL spectra of currently reported quasi-two-dimensional blue PeLEDs with superior electrical properties are generally in a relative long wavelength region [31]. By introducing a variety of macromolecular ligands, EQE values of 5.5% at 467 nm for quasi-two-dimensional blue perovskites and 6.3% at 471 nm for CsPb(Br_1−X_Cl_x_)_3_ quantum dots have been achieved. However, it is equally important to solve the spectral stability and to prepare a higher electrical performance for chlorine-doped blue LEDs [32,33]. In this work, perovskite films of CsPbCl_0.75_Br_2.25_ were obtained by introducing Cl^−^ into the lattice of CsPbBr_3_ through compositional engineering. Phenylethylammonium bromide (PEABr) was introduced into CsPbCl_0.75_Br_2.25_ perovskite films, and the perovskite films with better morphology and coverage were prepared by the ligand-assisted reprecipitation (LARP) method by dropping the anti-solvent at an optimal time. Referring to the in situ preparation of FAPbBr_3_ nanocrystals by Zhong’s group, we obtained the high luminescence and uniform perovskite nanocrystalline thin films [9]. Here, we use polar solvent dimethylsulfoxide (DMSO) to dissolve the perovskite precursor and chloroform as the anti-solvent to prepare excellent-performance CsPbCl_0.75_Br_2.25_ perovskite film. We have realized a blue emission efficient PeLED at 478 nm via employing the ITO/PEDOT: PSS/PVK/perovskite film/TPBi/LiF/Al device structure. The greatest EQE is 2.65% and the maximum brightness reaches 1069 cd/m^2^. Finally, the EL spectra of the chlorine-doped PeLEDs with PVK layer do not shift as the voltage increases.

## 2. Materials and Methods

### 2.1. Chemicals and Reagents

CsBr (99.999%), CsCl (99.999%) and PbBr_2_ (99.999%) were purchased from Sigma-Aldrich, St. Louis, MO, USA. PEABr (99.5%) was purchased from Xi’an Polymer Light Technology Corp., Xi’an, China, DMSO (>99.0%) and CHCl_3_ (>99.0%) were purchased from TCI, Gurgaon, India. LiF was purchased from Alfa Aesar, Haverhill, MA, USA. PEDOT: PSS (CH 8000) was purchased from Heraeus. TPBi was purchased from Lumtec. PVK was purchased from Lumtec.

### 2.2. Perovskite Solution Preparation

The CsPbCl_0.75_Br_2.25_ precursor solutions were prepared by dissolving 0.18 mmol CsCl, 0.06 mmol CsBr and 0.20 mmol PbBr_2_, and an appropriate amount of PEABr was dissolved in 1 mL DMSO under continuous stirring for 2 h at room temperature. The whole process of preparing the precursor solution was carried out in the glove box and the prepared raw materials were free of impurities. The ratio of x% PEABr refers to the molar ratio between PEABr and PbBr_2_ (i.e., mPEABr/mPbBr_2_ = x%), when x = 40, 60, 80, 100, the amount of PEABr, respectively, were 0.08 mmol, 0.12 mmol, 0.16 mmol and 0.20 mmol.

### 2.3. CsPbCl_0.75_Br_2.25_ Perovskite Nanocrystals (PNCs) Film Fabrication

All of the CsPbCl_0.75_Br_2.25_ PNCs films were fabricated on the PVK films. The different perovskite solution was spin-coated at 2500 rpm for 120 s and 200 uL trichloromethane was dropped on the spinning substrate after 30 s from the beginning of film rotation. In the process, the pipette head was completely perpendicular to the surface of the perovskite film. In order to prevent sputtering solution, the pipette head should be as close to the film as possible and should not touch the film surface. Then, it was dried at 80 °C for 5 min to remove residual solvent and ensure the complete reaction of the precursors. All of the above experiments were finished in a nitrogen filled glovebox.

### 2.4. LED Device Fabrication

The patterned ITO-coated glass substrates cleaned with detergent were sequentially cleaned in deionized water, acetone, ethanol, and deionized water by sonication for 15 min. After a 9 min O_2_ plasma treatment for ITO, PEDOT: PSS was spin-coated on the substrate at 3500 rpm for 60 s, followed by annealing at 150 °C for 15 min in ambient air. Then, PVK (10 mg/mL in THF) was spin-coated on the PEDOT: PSS film at 4000 rpm for 40 s and baked at 120 °C for 30 min. PVK film was then treated with O_2_ plasma for 15 min. Then, the substrates were transferred to a nitrogen-filled glovebox. Different DMSO precursor solutions were spin-coated on top of the PVK film to fabricate the CsPbCl_0.75_Br_2.25_ PNC films, as carried out previously. Finally, TPBi (20 nm), LiF (1 nm), and the Al electrode (100 nm) were deposited using a thermal evaporation system.

### 2.5. Device Measurements and Film Characterization

The morphology and energy-dispersive X-ray results of the perovskite films with different ratios of PEABr were obtained using field emission scanning electron microscopy (FE-SEM, Hitachi, Tokyo, Japan, SACS 4800). The voltage here is 5 KV, the working distance is 6900 um, and the emission current here is 1900 nA. A Nikon microscopy apparatus was applied to take the PL images of the perovskite films. Absorption and PL spectra at 360 nm excitation were evaluated at an indoor temperature under a xenon lamp at 150 W by a UV-3101 spectrophotometer and Hitachi F-7000 fluorescence spectrofluorometer, respectively. X-ray diffraction (XRD) patterns were characterized by the perovskite films by D8 Focus X-ray diffractometer purchased from Bruker Company. The 2θ Angle range of data acquisition during measurement was 10° to 80°, and 10° to 60° were captured for analysis. The Cu target was used as the radiation source. The radiation line Kα is λ = 1.54056 A. The operating voltage and current were 40 kV and 30 mA. The XRD step size was 0.02°, and the structure was confirmed to be a cubic crystal system by comparing with the standard card. Density—voltage characteristics, current efficiency—current density characteristics and power efficiency—voltage characteristics. The electroluminescence spectrum of the device was collected with Maya 2000 PRO spectrometer and the current-voltage-brightness and current-efficiency current-density characteristics of the device were tested by our self-built system. The test system includes: photometer, Geely voltage source and computer. A photometer (Konica Minolta LS-110, Tokyo, Japan) was used to record the brightness of the device. A Geely Time voltage source (Keithley 2400, Cleveland, OH, USA) acted as the voltage source of the device and tested the current flowing through the device. Finally, the software was used to read and record the brightness, voltage and current of the device, and calculate the current of the LED. The results of EQE were obtained from the system of the integrating sphere combined with the Ocean Optics spectrum-photometer.

## 3. Results

**Figure 1:**
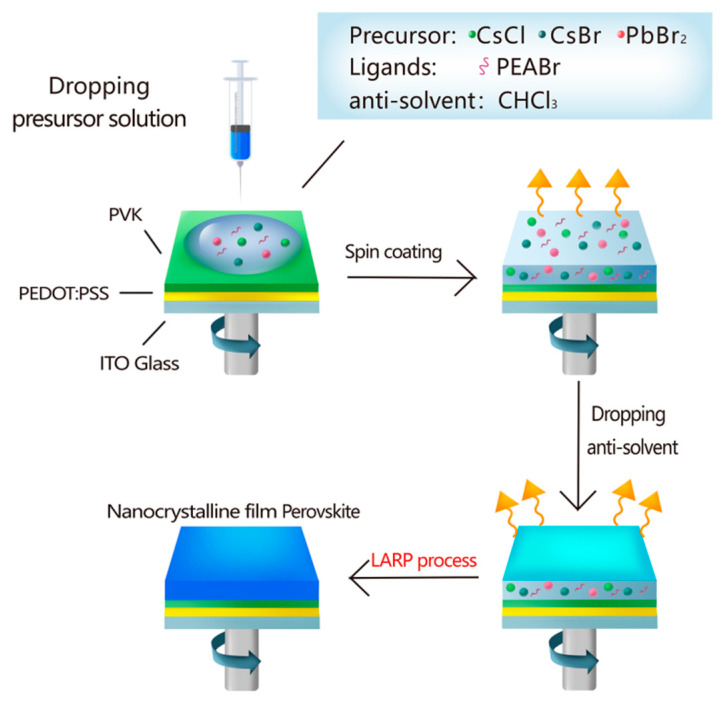
Schematic illustration of the in situ LARP to fabricateCsPbCl_0.75_Br_2.25_ PNC films.

Herein, a blue-emission perovskite film with CsPbCl_0.75_Br_2.25_ perovskite nanocrystals (PNCs) were prepared based on in situ LARP technology. Figure 1 schematically illustrates the in situ fabrication process of CsPbCl_0.75_Br_2.25_ PNC films. LARP has been demonstrated to be a versatile method to fabricate PNCs toward a highly efficient cladding solution by mixing the precursors in polar solvents with non-polar solvents. During the precipitation process, the change in solubility caused the nucleation process. The nucleation process resulted in differences in crystal size and crystal homogeneity compared with ordinary preparation methods. The in situ LARP method can typically produce smaller and more uniform crystals. Typically, in situ fabrication on a substrate for PeLEDs involves two steps: spin-coating a precursor solution in polar solvents (usually dimethylformamide, DMF or DMSO) and then dropping the anti-solvent at an appropriate time [9].

**Figure 2:**
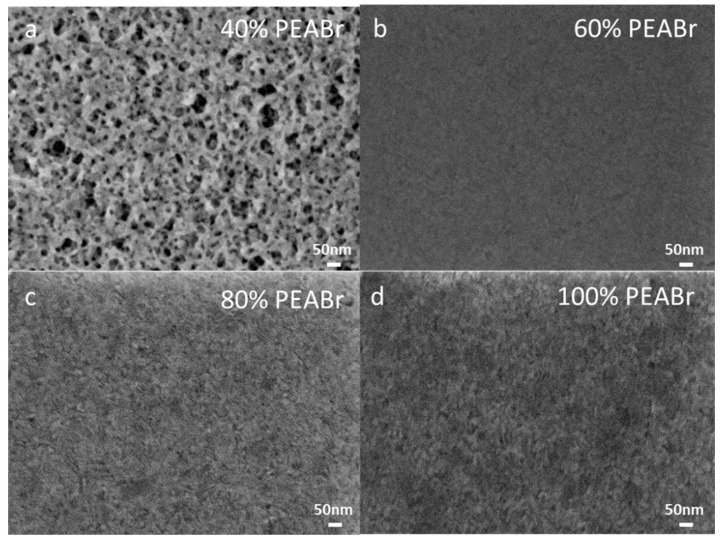
Top-view SEM images of perovskite thin films with (**a**) 40%, (**b**) 60%, (**c**) 80% and (**d**) 100% ratios of PEABr.

In order to obtain high-performance blue PeLEDs, high-quality and uniform-thickness CsPbCl_0.75_Br_2.25_ PNC films are essential. In this work, we introduced PEABr as the ligand for the preparation of perovskite films, and DMSO was selected as the solvent to disperse the perovskite precursor, and chloroform was selected as the anti-solvent to induce the reaction processes. The scanning electron microscope (SEM) images of the perovskite films with different ratios of PEABr including 40%, 60%, 80% and 100% are shown in Figure 2. The SEM images of the perovskite films without PEABr are shown in Figure S1. With the increase in the amount of PEABr to 60%, a regular perovskite small crystal was obtained. Moreover, the perovskite film became more uniform and denser. This means that the introduction of PEABr can effectively regulate the formation of perovskite films. However, with the continuous increase in the amount of macromolecular ligand PEABr to 100%, the excessive addition of PEABr led to phase separation between PEABr and perovskite. The perovskite crystal returned to its irregular shape, resulting in a poorer crystal quality perovskite film [10,16]. The film quality can also be observed from the comment spectra. As shown in Table 1, the films with 60% PEABr had the strongest emission intensity and the smallest FWHM. This indicates that the nanocrystal size of the film was the most uniform, the film was well passivated and the occurrence of non-radiative recombination was weakened. As shown in Table S1 (Supplementary Material), the Photoluminescence quantum yield (PLQY) of the film was also enhanced with the increase in PEABr and then weakened due to the excess of PEABr.

**Figure 3:**
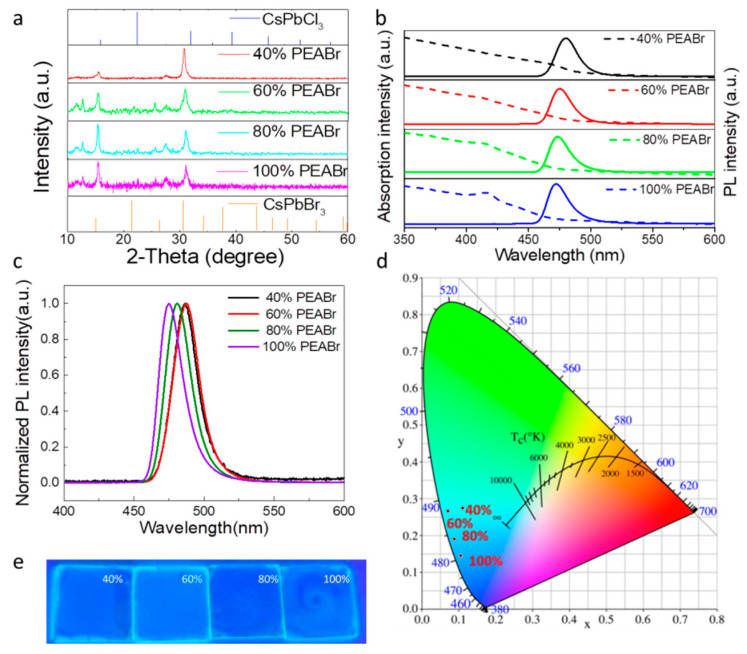
(**a**) XRD patterns, (**b**) absorption and PL spectra, (**c**) normalized PL spectra, and (**d**) corresponding CIE of CsPbCl_0.75_Br_2.25_ thin films with different ratios of PEABr. (**e**) Images of the blue perovskite films with different ratios of PEABr under 365 nm light irradiation.

The structure and properties of the perovskite films have been studied by X-ray diffraction (XRD), as shown in Figure 3a. In the original CsPbCl_0.75_Br_2.25_ film, a main peak appeared at 2θ angle of 15.4°, corresponding to the (100) plane of the 3D perovskite. A main peak appeared at 2θ angle of 30.8°, corresponding to the (200) plane of the 3D perovskite. With the increase in PEABr content, the crystal peak intensity of (200) decreased, while that of (100) increased. These results indicate that the PEABr can induce CsPbCl_0.75_Br_2.25_ perovskite crystal to grow oriented in (100) direction. The perovskite film was identified as CsPbBr_3_ and CsPbCl_3_ according to the standard card. As shown in Figure S4, we calculated and measured the lattice sizes of 0.290 nm and 0.283 nm in TEM images, corresponding to the (200) sides of the CsPbBr_3_ (0.2915 nm). Additionally, the lattice sizes of 0.231 nm corresponded to the (211) sides of the CsPbBr_3_ (0.238 nm) and CsPbCl_3_ (0.2288 nm). The absorption and PL spectra were measured and are shown in Figure 3b. It can be implied that with the increase in the amount of PEABr, the absorption and PL peaks gradually shifted to a short wavelength. The same trend based on the normalized PL measurements can be found in Figure 3c. With the increase in the proportion of perovskite generation, the wavelength of the PL peak blue shifted from 488 nm to 475 nm. It is worth noting that there should be several specific emission peaks if they were composed of different types of 2D perovskites, as reported in the previous work [31]. Here, the PL peaks present significant Gaussian distribution without the characteristic peaks of two-dimensional perovskites. From Figure 3d, the corresponding Commission Internationale de L’E chlaiage (CIE), Vienna, Austria coordinates agree well with the color change. The images of the blue perovskite films with the amount of PEABr ligand, increased from 40% to 60%, 80% and 100% under 365 nm light irradiation, are shown in Figure 3e. The colors of the perovskite films are obviously blue; moreover, the chemical composition of the synthesized CsPbCl_0.75_Br_2.25_ PNCs was analyzed via energy dispersive spectrometer (EDS) spectroscopy. As shown in Figure S2 (Supplementary Material) and Figure S3 (Supplementary Material), elements of Br, Pb, Cl and Cs are confirmed, indicating CsPbCl_0.75_Br_2.25_. As shown in Table S3 (Supplementary Material), the atom molar ratio of Cl to Br is about 1:3, which fits to the data of our pre-design CsPbCl_0.75_Br_2.25_ PNCs. These results demonstrated the successful synthesis of the CsPbCl_0.75_Br_2.25_ PNCs via our method.

**Figure 4:**
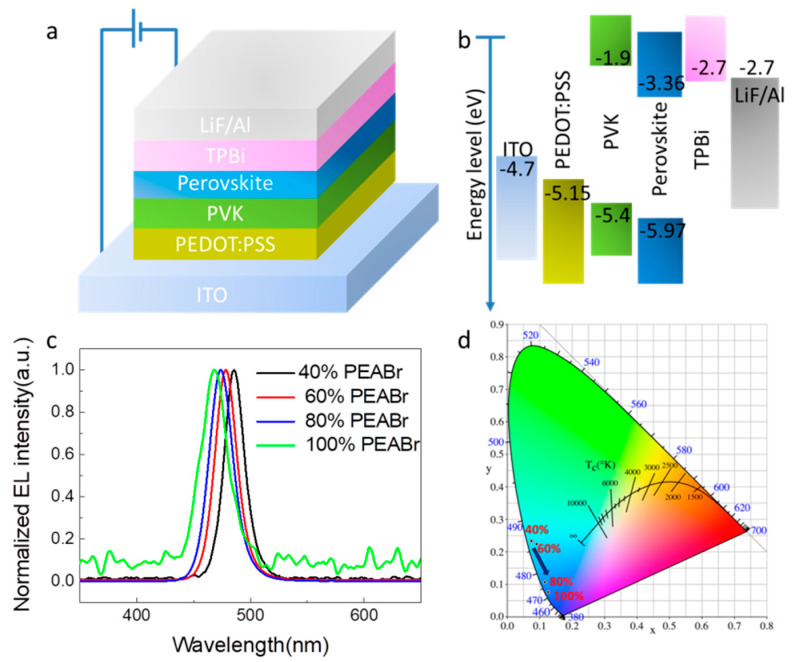
(**a**) PeLED architecture. (**b**) Energy levels for different layers of the device. (**c**) EL spectra of PeLEDs with different ratios of PEABr. (**d**) CIE values of the EL spectra of PeLEDs.

**Figure 5:**
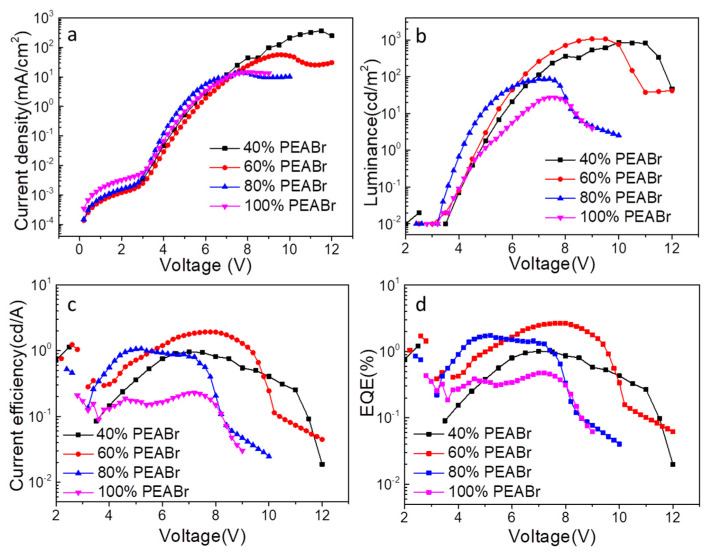
Performance of CsPbCl_0.75_Br_2.25_ PeLEDs with different ratios of PEABr. (**a**) current density versus voltage, (**b**) luminance versus voltage, (**c**) current efficiency versus voltage, and (**d**) EQE versus voltage curves.

Encouraged by the above results, we further fabricated the blue PeLEDs using perovskite films with different ratios of PEABr and a device structure of ITO/PEDOT: PSS/PVK/perovskite film/TPBi/LiF/Al, as indicated in Figure 4a. Their corresponding energy levels of the composed layers are shown in Figure 4b. The normalized EL spectrum of the blue PeLEDs by adding different ratios of PEABr were detected under 6 V, as shown in Figure 4c. The results showed that with the increase in PEABr content, the peak of the EL spectrum shifted from 485 nm to 468 nm, which was closely consistent with the results of the PL spectra. With the blue shift in EL spectra, the corresponding CIE value also changed from the sky-blue to pure-blue region, which meets the requirement of broad color gamut display. Figure 5 and Table 2 summarize the electrical properties of the devices prepared with different proportions of PEABr CsPbCl_0.75_Br_2.25_ thin films. It can be seen from Figure 5b that the working voltage of PeLED with different proportions of PEAB can be at least about 4 V, which conforms to the condition of the low working voltage of the device, indicating that the balance of PeLED carrier injection is excellent. We found that higher brightness (1069 cd/m^2^, Figure 5b, Table 2) was achieved through 60% PEABr addition. The best EQEs and current efficiency (CE) of 2.65% and 1.92 cd/A were found in device with 60% PEABr. The EL properties of the perovskite films are closely related to the trap density of the perovskite film. The higher the trap density is, the more severe traps, resulting in more severe non-radiation trap-assisted recombination. Due to the large number of pinholes in the pristine perovskite film, the introduction of ligand PEABr led to a better coverage and crystal quality. In other words, the introduction of 40% and 60% PEABr can effectively inhibit the current leakage. However, when the concentration of PEABr is further increased to 80% or 100%, the crystal quality of the thin films decreases compared with that of 60% PEABr. This is because excessive PEABr leads to phase separation with perovskite, so the PeLED performance also decreases.

**Figure 6:**
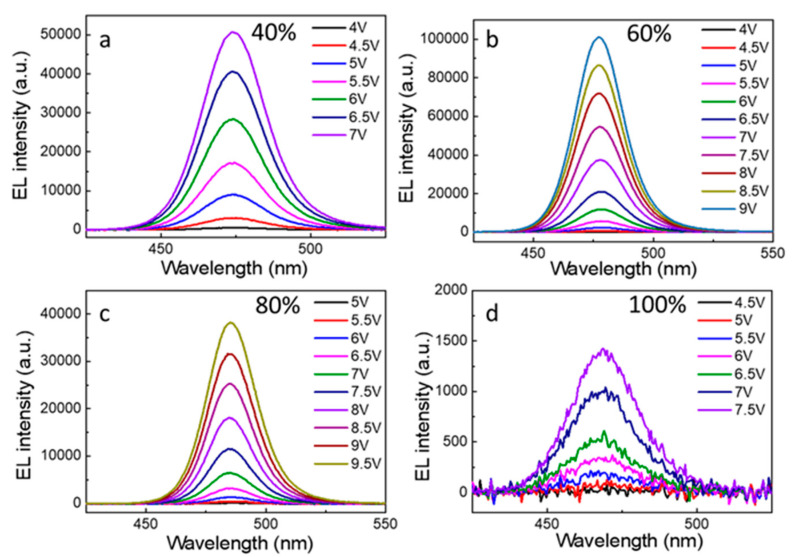
EL spectra of PeLEDs with (**a**) 40%, (**b**) 60%, (**c**) 80% and (**d**) 100% ratios of PEABr.

When the content of PEABr is 60%, the perovskite film had the most excellent film morphology. The SEM and transmission electron microscope (TEM) of the superior morphological information is shown in Figure 2b and Figure S4 (Supplementary Material). We can see that the dimeter of the CsPbCl_0.75_Br_2.25_ PNCs is about 10 nm from Figure S4 (Supplementary Material). Figure S3c (Supplementary Material) and Figure S3d (Supplementary Material) show the spacings of the CsPbCl_0.75_Br_2.25_ PNCs, which is consistent with the previously measured XRD performance. Based on the high-quality film morphology, the prepared blue LED device has the superior electrical properties, which is shown in Figure 5. We measured the values of current density versus voltage in Figure 5a. As shown in Figure 6b, we observed 8.8 V, as shown in Figure 5c. From Figure 5d, we can calculate the EQE of the LED devices, in which the largest value is 2.65% on the voltage of 8.8 V. These results demonstrated the blue emitting PeLED has the predominant electrical properties. The blue emitting PeLED is shown in Figure S5 (Supplementary Material). Last but not least, the blue emitting PeLED still has a relatively high value of EQE on a comparatively high voltage.

Figure 6 shows the EL spectra of PeLEDs with different PEABr content under different operating voltages obtained recorded by a fiber optic spectrometer. It is notable that the position of the EL peak does not shift with the increase in voltage. Even at a driving voltage of 9 V, the device still shows good spectral stability. Figure S6 (Supplementary Material) shows that the EL spectra of ordinary devices without a PVK layer were significantly red shifted with increasing voltage, which is attributed to the perovskite phase with large n induced by PEDOT: PSS layer [31]. We also present the max EL intensity comparison of 60% PEABr at different voltages with or without the PVK layer in Table S3 (Supplementary Material). The electroluminescence performance of the PeLED with the PVK layer is much better than that without the PVK layer, especially at high pressure. The introduction of the PVK hole transport layer in the device can effectively isolate the PEDOT: PSS layer and luminescence layer, which can reduce or even eliminate the adverse induction of the PEDOT: PSS layer to the luminescence layer and successfully inhibit halide atom migration. In addition, the PVK layer with hydrophobic properties can provide environmental stability and effectively inhibit the non-radiative recombination of the metal halide luminescent layer. Combined with the high-quality thin film prepared by in situ LARP method such as luminescent layer, the PeLEDs finally shows excellent spectral stability.

## 4. Conclusions

In summary, we have realized stable and efficient blue PeLED emission at 478 nm using the ligand-assisted reprecipitation method. The maximum brightness is 1069 cd/m^2^ and the greatest EQE is 2.65%. The introduction of PEABr can effectively passivate the defect states on the surface of the perovskite film, making the perovskite film more compact. With the increase in PEABR, the PL peaks shift to a short wavelength. By introducing the PVK hole transport layer, the device can obtain efficient hole injection. The PVK layer with hydrophobic properties can provide environmental stability and can effectively isolate the PEDOT: PSS layer and luminescence layer and, thus, inhibit halide atom migration. Consequently, the EL peaks of the devices made by our method do not shift as the voltage increases. In other words, the devices have suitable spectral stability even at a high voltage, which is attributed to the combined effect of the PEABr and PVK layer. This overcomes the fatal shortcoming of voltage instability caused by an unstable power spectrum and greatly improves the application value and prospect of blue PeLEDs.

## Figures and Tables

**Table 1:** Summary of CsPbCl_0.75_Br_2.25_ perovskite films with different ratios of PEABr.

	PL (nm)	The Max PL Intensity	FWHM (nm)
40% PEABr	480	33,886	21.8
60% PEABr	475	56,537	20.4
80% PEABr	473	49,984	20.7
100% PEABr	472	22,316	22.6

**Table 2:** Summary of CsPbCl_0.75_Br_2.25_ PeLEDs with different ratios of PEABr.

	CE (cd/A)	Luminance (cd/m^2^)	EQE	EL (nm)	FWHM (nm)
40% PEABr	0.95	846.9@10 V	1.01@7 V	485	22
60% PEABr	1.92	1069@9 V	2.65@8.8 V	478	23
80% PEABr	1.06	86@7.4 V	1.73@5.2 V	474	24
100% PEABr	0.23	27.3@7.6 V	0.47@7 V	468	26

## Data Availability

Not applicable.

## Supplementary Materials

The following are available online at https://www.mdpi.com/article/10.3390/nano12172906/s1, Figure S1: The SEM images of the perovskite films without PEABr; Figure S2: Energy dispersive spectrometer (EDS) spectrum of the CsPbCl_0.75_Br_2.25_ PNCs with different ratios of PEABr; Figure S3: Energy dispersive spectrometer mapping image of the CsPbCl_0.75_Br_2.25_ PNCs with 60% ratios of PEABr; Figure S4: top-view transmission electron microscope (TEM) images of perovskite thin films with 60% ratios of PEABr; Figure S5: CsPbCl_0.75_Br_2.25_ PeLEDs with 60% ratios of PEABr in in daylight and the darkness; Figure S6: The EL spectra of the PeLEDs without PVK layer and with 60% ratios of PEABr; Table S1: The optical properties of CsPbCl_0.75_Br_2.25_ films with different ratios of PEABr; Table S2: Element composition of Br, Pb, Cl and Cs atoms of the CsPbCl_0.75_Br_2.25_ PNCs samples calculated by the EDS results from Figure S1; Table S3: The max EL intensity comparison of 60% PEABr at different voltages with or without PVK layer.
