# Supplementary material for: Spectrally Stable Blue Light-Emitting Diodes Based on All-Inorganic Halide Perovskite Films
Source: Nanomaterials (Basel). 2022 Aug 24;12(17):2906. doi: 10.3390/nano12172906 (PMC9457983; doi:10.3390/nano12172906)
Supplement: Supplementary file 1 [file nanomaterials-12-02906-s001.zip › nanomaterials-1816832-supplementary.pdf]

Supplementary Materials

Spectrally Stable Blue Light-Emitting Diodes Based on All-Inorganic Halide Perovskite Films

Huidan Zhang [1,2,3], Ying Su [1,4,*], Xulan Xue [5], Qinghui Zeng [1,3,*], Yifang Sun [1,2], Kai Zhu [3], Weiguang Ye [1], Wenyu Ji [5] and Xiangyang Leng [3,*]

[1] State Key Laboratory of Luminescence and Applications, Changchun Institute of Optics, Fine Mechanics and Physics, Chinese Academy of Sciences, Dong_Nanhu Road 3888, Changchun 130033, China; zhanghuidan18@mails.ucas.ac.cn (H.Z.); sunyifang18@mails.ucas.ac.cn (Y.S.); yeweiguang130391@sina.com (W.Y.)

[2] University of Chinese Academy of Sciences, Beijing 100049, China

[3] Changchun University of Chinese Medicine, Changchun 130017, China; zhukai@ccucm.edu.cn

[4] School of Optoelectronic Engineering and Instrumentation Science, Dalian University of Technology, Dalian 116024, China

[5] Key Lab of Physics and Technology for Advanced Batteries (Ministry of Education), Department of Physics, Jilin University, Changchun 130012, China; xuexl20@mails.jlu.edu.cn(X.X.); jiwy@jlu.edu.cn (W.J.)

* Correspondence: suying9204@163.com (Y.S.); zengqinghui96000@163.com (Q.Z.); lengxiangy@163.com (X.L.);
Tel.: 15526635564(Y.S.); +86-0431-86708506 (Q.Z.)

Table S1. The optical properties of $CsPbCl_{0.75}Br_{2.25}$ films with different ratios of PEABr. The value of PLQY can be compared with the ratio of the PL peak area and the Absorption at 360nm

	PL peak area	The Absorption at 360nm	PL peak area/ The Absorption at 360nm
40%PEABr	3,377,251	0.2300	$1.468*10^7$
60%PEABr	3,861,481	0.2054	$1.880*10^7$
80%PEABr	3,790,509	0.1809	$2.095*10^7$
100%PEABr	3,163,313	0.1661	$1.904*10^7$

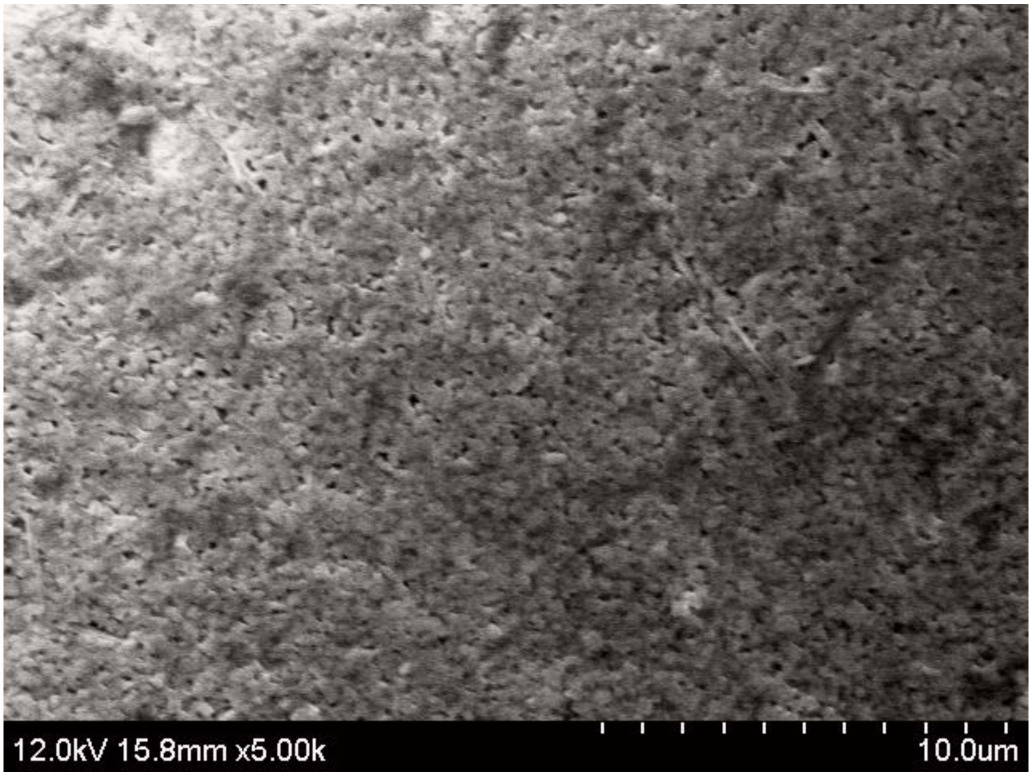

Figure S1. The SEM images of the perovskite films without PEABr.

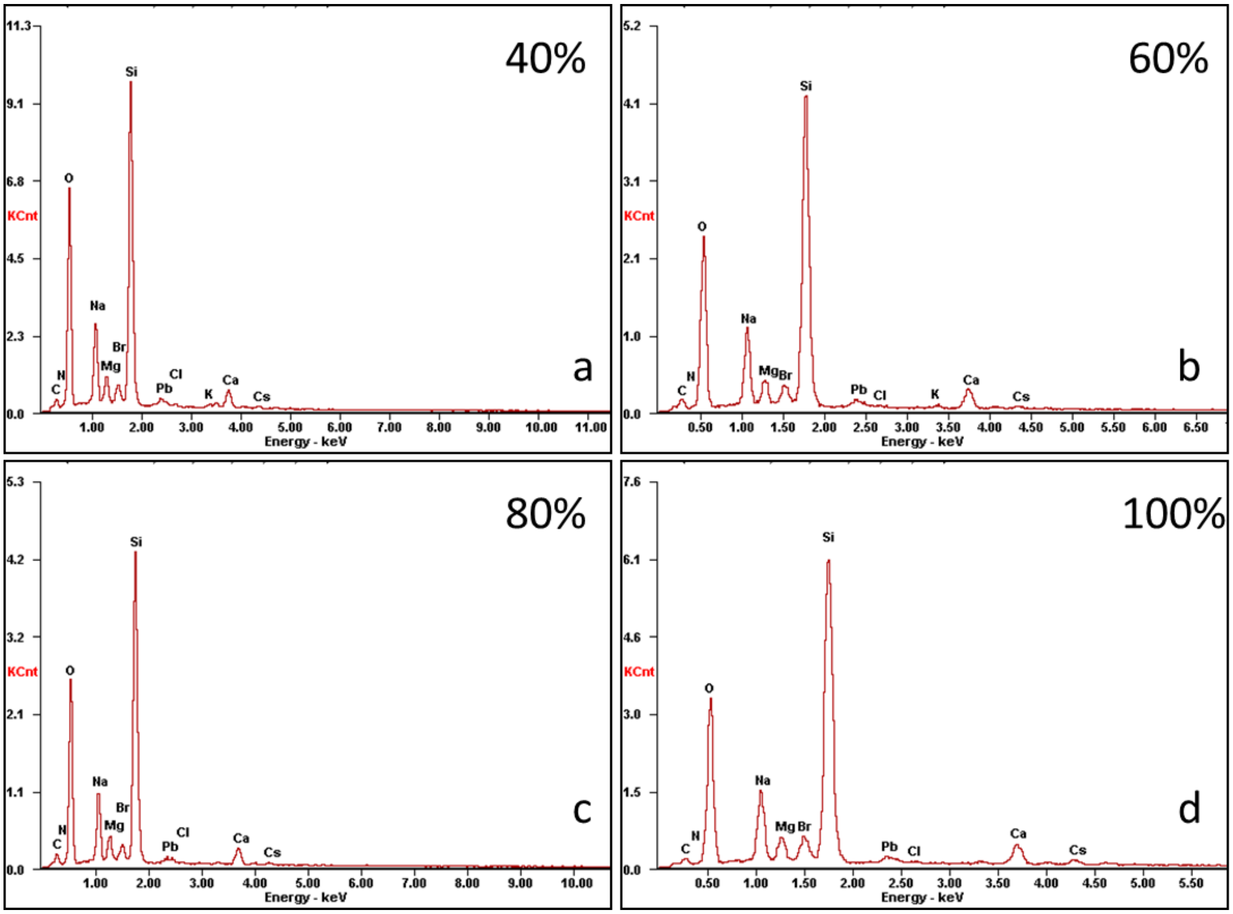

Figure S2. Energy dispersive spectrometer (EDS) spectrum of the $CsPbCl_{0.75}Br_{2.25}$ PNCs with different ratios of PEABr.

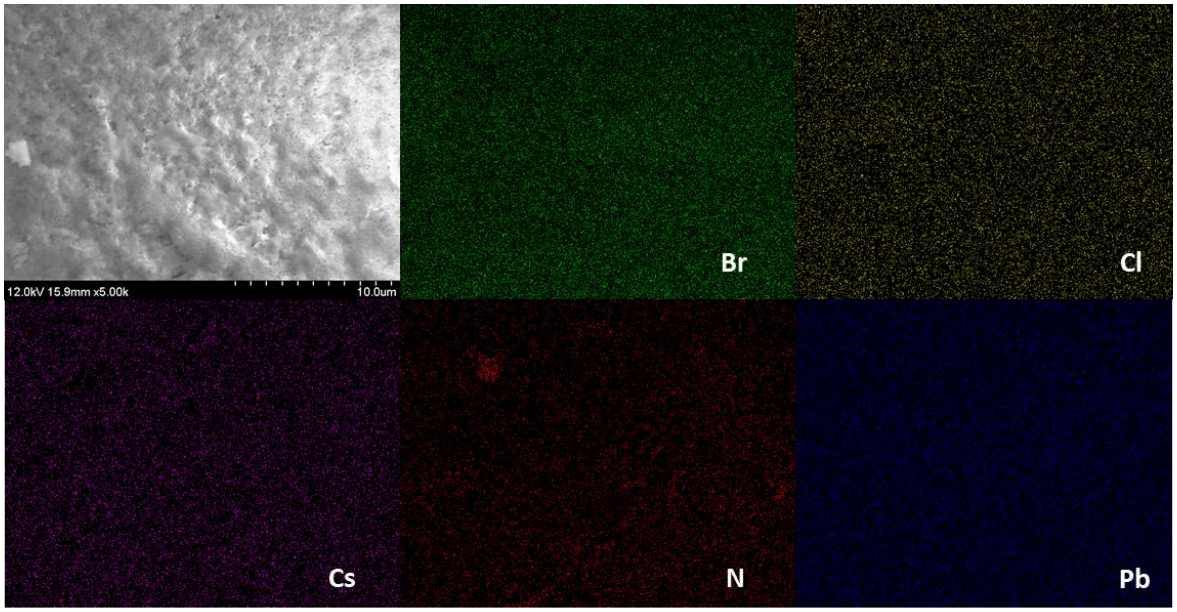

Figure S3. Energy dispersive spectrometer mapping image of the $CsPbCl_{0.75}Br_{2.25}$ PNCs with 60% ratios of PEABr.

Table S2. Element composition of Br, Pb ,Cl and Cs atoms of the $CsPbCl_{0.75}Br_{2.25}$ PNCs samples calculated by the EDS results from Figure S1.

PEABr	Element	Wt %	At %
40%	BrL	29.74	14.65
	PbM	26.13	04.96
	ClK	02.95	03.27
	CsL	15.34	04.54
60%	BrL	31.64	18.89
	PbM	27.29	06.28
	ClK	03.35	04.51
	CsL	19.10	06.86
80%	BrL	35.18	18.05
	PbM	24.71	04.89
	ClK	02.44	02.83
	CsL	13.77	04.25
100%	BrL	35.86	22.11
	PbM	20.87	04.96
	ClK	02.35	03.27
	CsL	23.60	08.75

Table S3. The max EL intensity comparison of 60% PEABr at different voltages with or without PVK layer

	The max EL intensity(5.5V)	The max EL intensity(6.5V)	The max EL intensity(7.5V)
Without PVK layer	9804	16818	9278
With PVK layer	5832	21005	54637

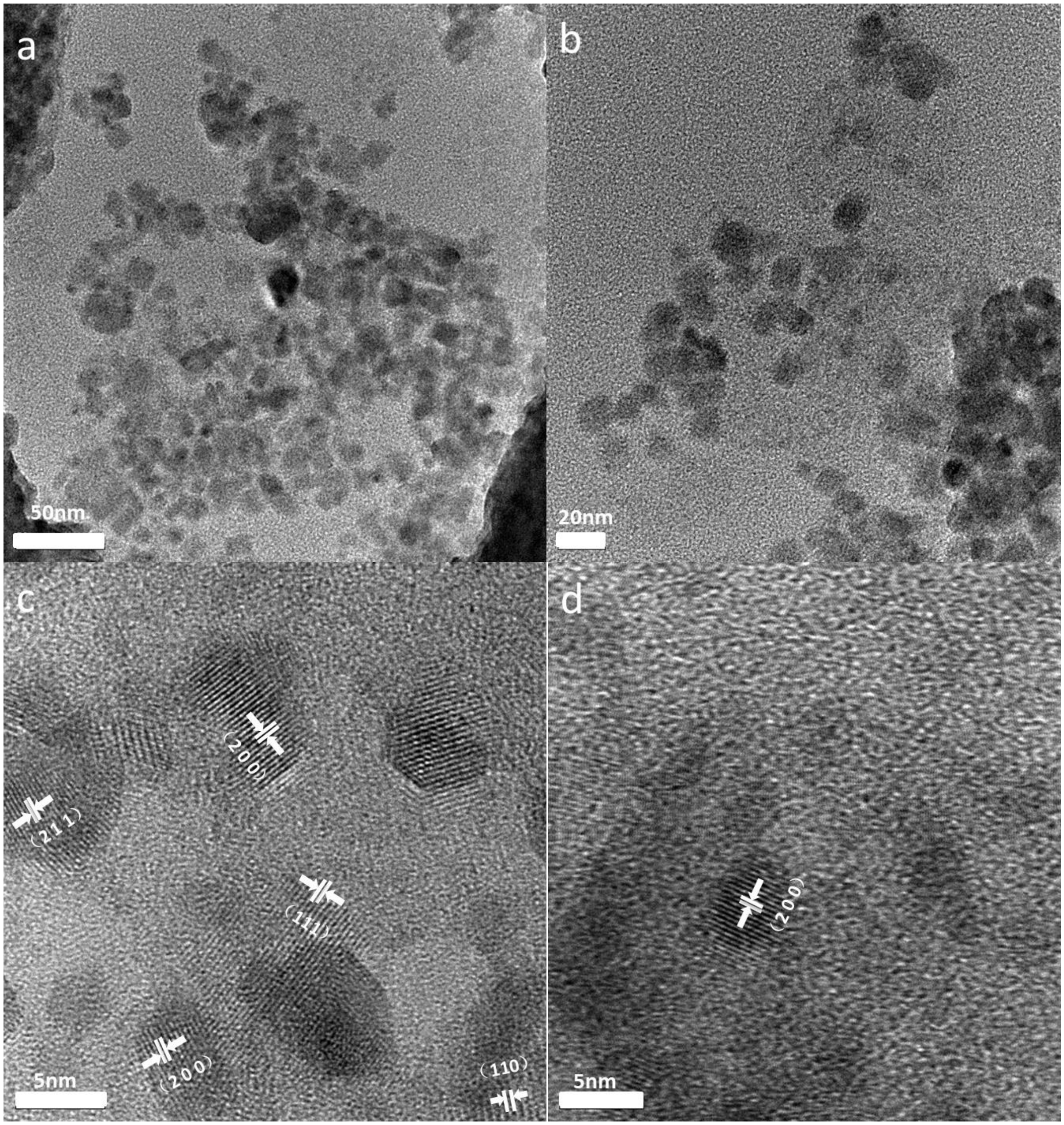

Figure S4. top-view transmission electron microscope (TEM) images of perovskite thin films with 60% ratios of PEABr.

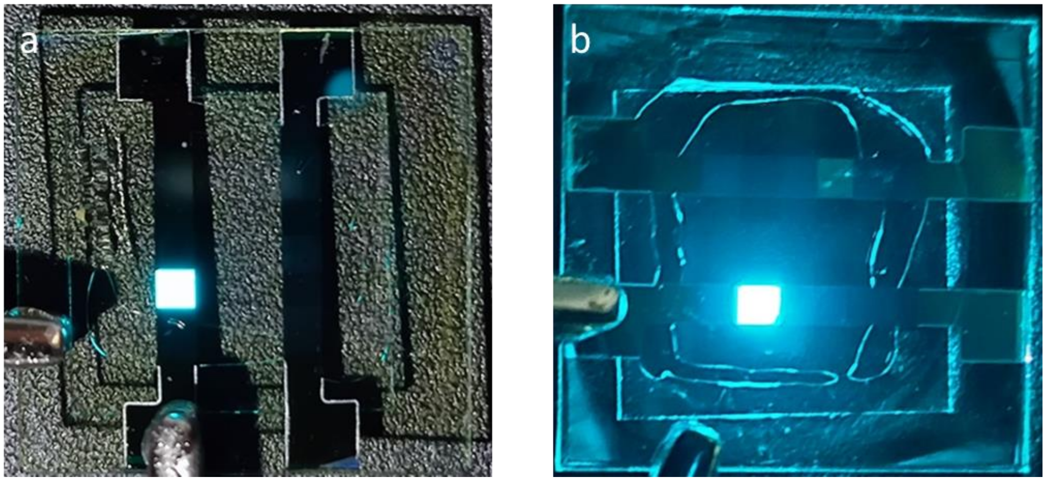

Figure S5. $CsPbCl_{0.75}Br_{2.25}$ PeLEDs with 60% ratios of PEABr in a) daylight and b) the darkness.

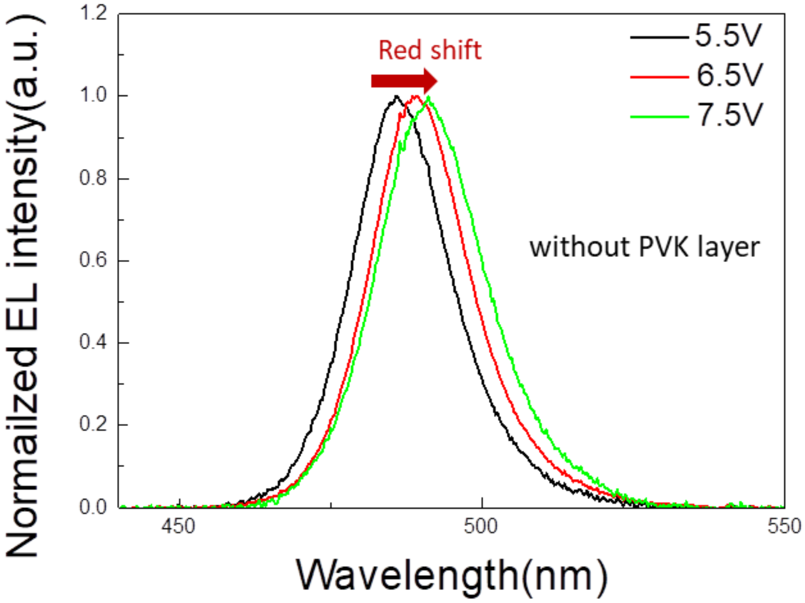

Figure S6. The EL spectra of the PeLEDs without PVK layer and with 60% ratios of PEABr.

The chemical composition of the synthesized $CsPbCl_{0.75}Br_{2.25}$ PNCs was analyzed via energy dispersive spectrometer (EDS) spectroscopy. As shown in Figure S1 and Figure S2, elements of Br, Pb, Cl and Cs are confirmed, indicating the formation of $CsPbCl_{0.75}Br_{2.25}$. As shown in Table S1, the atom molar ratio of Cl to Br is about 1:3, which is fit to the data of our pre-design $CsPbCl_{0.75}Br_{2.25}$ PNCs. These results demonstrated the successful synthesis of the $CsPbCl_{0.75}Br_{2.25}$ PNCs via our method.
